# Universality of light thermalization in multimoded nonlinear optical systems

**DOI:** 10.1038/s41467-023-35891-9

**Published:** 2023-01-23

**Authors:** Qi Zhong, Fan O. Wu, Absar U. Hassan, Ramy El-Ganainy, Demetrios N. Christodoulides

**Affiliations:** 1 CREOL, College of Optics and Photonics, University of Central Florida, Orlando, FL 32816 USA; 2 Department of Physics, Michigan Technological University, Houghton, MI 49931 USA; 3 Henes Center for Quantum Phenomena, Michigan Technological University, Houghton, MI 49931 USA; 4 Ming Hsieh Department of Electrical and Computer Engineering, University of Southern California, Los Angeles, CA 90089 USA

**Keywords:** Nonlinear optics, Optical physics

## Abstract

Recent experimental studies in heavily multimoded nonlinear optical systems have demonstrated that the optical power evolves towards a Rayleigh–Jeans (RJ) equilibrium state. To interpret these results, the notion of wave turbulence founded on four-wave mixing models has been invoked. Quite recently, a different paradigm for dealing with this class of problems has emerged based on thermodynamic principles. In this formalism, the RJ distribution arises solely because of ergodicity. This suggests that the RJ distribution has a more general origin than was earlier thought. Here, we verify this universality hypothesis by investigating various nonlinear light-matter coupling effects in physically accessible multimode platforms. In all cases, we find that the system evolves towards a RJ equilibrium—even when the wave-mixing paradigm completely fails. These observations, not only support a thermodynamic/probabilistic interpretation of these results, but also provide the foundations to expand this thermodynamic formalism along other major disciplines in physics.

## Abstract (web summary)

The origin of the Rayleigh–Jeans distribution associated with light thermalization in optical thermodynamic multimode nonlinear settings is discussed. Here the authors show that due to entropy maximization, this process is universal and is independent of the intricacies of the nonlinearities involved.

## Introduction

Nonlinear optics plays a crucial role in a wide range of modern science and technologies. These include optical cavity microcombs^1,2^, high-power light sources^3^, cavity optomechanics^4,5^, nonlinear topological and non-Hermitian photonics^6–10^, bioimaging^11,12^, as well as classic/quantum networks and signal processing^13–16^. While nonlinear interactions widely vary in strength and differ from one material system to another, their vast majority can still be described using an underlying theoretical framework that relies on perturbative analysis^17^. Particularly, by expressing the electric polarization vector as a Taylor series expansion in terms of the driving electric field, one can classify nonlinear optical effects into several, largely independent processes such as those associated with second harmonic and sum/difference frequency generation and multi-wave mixing interactions^17^. A few decades ago, this same paradigm was adopted by Zakharov and colleagues to study optical nonlinear propagation effects when an infinite number of Fourier components is involved—a field of research that is nowadays known as wave turbulence^18^. In this seminal work, it was shown that such a system can be described by a Boltzmann-like kinetic model that admits a steady-state solution in the form of a Rayleigh–Jeans (RJ) distribution. In this regard, it was conjectured that the RJ law results as a mere byproduct of the nonlinear attractor dynamics taking place during multi-wave mixing^19^. In developing this model, several assumptions were made. Firstly, it was implicitly assumed that four-wave mixing dominates the interaction process. Secondly, the so-called random phase approximation^20^ was employed to omit off-resonant interaction terms. Meanwhile, recent progress in the general area of multimode fiber optics^21–29^ has enabled a new generation of nonlinear experimental setups where the RJ distribution (power allocation among modes) was successfully observed for the first time^30–33^. The clear demonstration of RJ thermalization in such settings has been touted as evidence in support of the wave turbulence theory. While reaching such a conclusion does not seem to pose a problem from a practical point of view, it is unsettling at a more fundamental level. In essence, adopting the wave turbulence hypothesis is to a great extent analogous to attempting to infer, for example, the nature of the interactions between gas molecules solely from the Maxwell–Boltzmann distribution. Even more importantly, while the laws of simple thermodynamic systems like gases can be developed from either classical (Newtonian) kinetic theories or quantum mechanical perspectives, this is by no means necessary, given that the corresponding equations of state can be derived from purely entropic principles—in total disregard to the underlying collisional mechanisms. So, the question naturally arises: is the RJ distribution an actual byproduct of multi-wave mixing processes or does it represent a much more general result that has little to do with the specifics of the inherent nonlinearity involved?

**Fig. 1:**
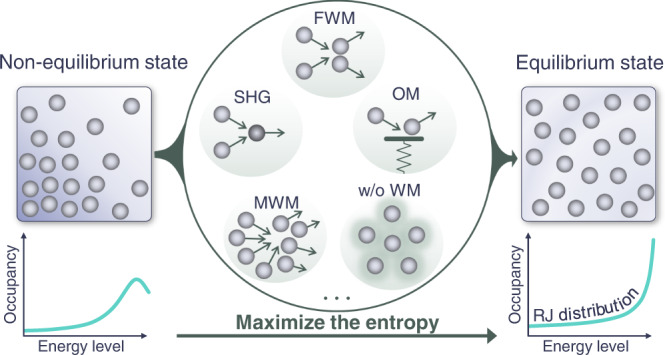
Conceptual illustration of thermalization in a nonlinear multimode optical system. Similar to thermalization in matter, the nature of the interaction forces (like forces between gas molecules) is irrelevant. Here, we show that light thermalization into a Rayleigh–Jeans (RJ) distribution can take place under a wide range of nonlinear conditions beyond the traditional four-wave mixing (FWM) paradigm. These include second harmonic generation (SHG), multi-wave mixing (MWM), optomechanical (OM) cascaded interactions between optical and mechanical modes, and even scenarios where the system cannot be described by any wave mixing expansion (w/o WM).

Quite recently, a different approach for studying light thermalization was put forward on the basis of statistical mechanics and thermodynamics^34–38^. While this latter theoretical framework reaches similar conclusions to those derived from the aforementioned kinetic theories^18,19^ as far as the RJ distribution is concerned, its perspective of optical thermalization is fundamentally different. Being founded on notions from statistical mechanics, this paradigm^34,35^ allows one to predict and interpret the RJ law emerging in a microcanonical system from purely entropic considerations. In this regard, the RJ equilibrium state macroscopically manifests itself because it is ergodically associated with a largest number of microstates (in phase space) and thus it can be considered a byproduct of probability theory—an aspect that has little to do with the nature of the underlying nonlinearity involved. If this is indeed the case, then in analogy with statistical mechanics of gases, the RJ thermalization should occur in systems with more generic nonlinearities beyond the wave mixing paradigm as illustrated in Fig. 1. The situation is however more complex. Nonlinear optical systems often exhibit two constants of motion, i.e., the power and the Hamiltonian. The first, which describes the conservation of optical power, is analogous to the number of particles in a gas system. The second, however, when expressed in the linear eigenbasis, involves both a linear and a nonlinear component. Thus, strictly speaking, such a system is not necessarily expected to relax to a RJ distribution. Only under the condition that the linear part is constant, the RJ distribution can be anticipated. In reality, however, even under weak nonlinear conditions, the linear part of the Hamiltonian is only quasi-conserved. In other words, the analogy between multimoded nonlinear optical arrangements and idealized thermodynamic systems involving two constants of motion is not formal, which further complicates the question about thermalization in nonlinear optical systems and the physical mechanism responsible for observing the RJ distribution.

In this work, we critically examine the manner in which optical thermalization processes unfold in nonlinear environments with different types of nonlinearities such as those arising from optomechanical interactions (where wave mixing interpretations are rather cumbersome) and those associated with photorefractive crystals (where above certain power thresholds, standard perturbative wave mixing expansions are not possible). In addition, we consider also artificial nonlinear systems with nonanalytic and discontinuous nonlinear functions that cannot be described by any convergent polynomial and demonstrate that such set-ups can also reach the RJ equilibrium distribution. Our work thus establishes the universality of the thermalization towards the RJ state in nonlinear optical systems, and, in doing so, presents compelling evidences in favor of the more general entropic view of optical thermalization as opposed to the more restrictive four-wave mixing paradigm.

## Results

Before we proceed, perhaps it would be useful to highlight some of the basic notions upon which the optical thermodynamic approach relies on. As in the case of standard statistical mechanics^39^, the entropy of the optical multimode arrangement can be built within a microcanonical ensemble formalism by accounting all possible microstates, each containing information as to the energy/power and phase distribution among all modes in the system. In defining the macrostates, the energy/power distribution is retained while the phase information is omitted^40^ (being superfluous given that it is uniformly distributed within the range 0 to 2*π*). In this respect, the nonlinear interaction acts merely as an agent that enables a chaotic reshuffling of optical energy among modes and therefore facilitates thermalization. On the other hand, the specifics of nonlinearity are inconsequential. Optical thermodynamic equilibrium is then reached when entropy is maximized over all possible microstates under the constraints dictated by the two constants of motion^35^.

### Kerr nonlinearity

**Fig. 2:**
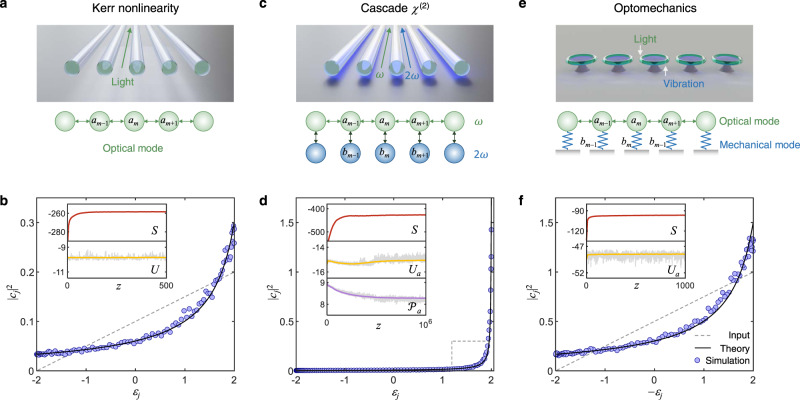
Thermalization of light in nonlinear waveguide arrays with different nonlinearities. Linear and nonlinear couplings in three optical lattices when acted upon by three different nonlinearities: **a** a Kerr nonlinearity, **c** cascade *χ*^(2)^ process, and **e** optomechanical nonlinearities, as described by Eqs. (1), (3), and (4), respectively. Numerical simulations provide the modal occupancies after thermalization in all these three scenarios, in good agreement with the predicted Rayleigh–Jeans (RJ) distributions (black lines), as shown in **b**, **d**, and **f**. The insets display a monotonic increase in entropy *S* as well as the invariants of the motion *U* and ${{{{{{{\mathcal{P}}}}}}}}$. Note that in all cases, numerical simulations are performed over ensemble averages. The thermal fluctuations of quasi-invariants (when applicable) are indicated by gray lines, depicting the instantaneous values of *U* and ${{{{{{{\mathcal{P}}}}}}}}$ around their mean values. In all cases, the nonlinear array has *M* = 100 sites and the dashed lines represent the initial occupancies for the linear optical supermodes.

We begin our analysis by first considering a Kerr nonlinear multimode tight-binding model—a one-dimensional photonic array comprised of *M* evanescently coupled single-mode waveguides with nearest neighbor coupling^41,42^ (a situation most relevant to experimental implementations), as shown in Fig. 2a. Under these conditions, light propagation along *z* in such a lattice can be described by the following normalized discrete nonlinear Schrödinger equation^43^:

1 $$i\frac{d{a}_{m}}{dz}+{a}_{m-1}+{a}_{m+1}+|{a}_{m}{|}^{2}{a}_{m}=0,$$

where *a*_*m*_ is the field amplitude at site *m*, and the last term denotes Kerr nonlinear effects. Equation (1) exhibits two constants of motion. The first invariant (denoting power conservation) is given by ${{{{{{{\mathcal{P}}}}}}}}=\mathop{\sum }\nolimits_{m=1}^{M}|{a}_{m}{|}^{2}=\mathop{\sum }\nolimits_{j=1}^{M}|{c}_{j}{|}^{2}$, where *c*_*j*_ is the field amplitude component associated with supermode $|{\psi }_{j}\rangle$ of the linear array (i.e., the normal modes obtained by diagonalizing Eq. (1) in the absence of the nonlinear term). The complex amplitudes *c*_*j*_ at any distance *z* are obtained by projecting the state $\left|\psi \right\rangle$ of the system on the linear supermodes as expressed in the local representation (i.e., in terms of *a*_*m*_). The second invariant is associated with the optical Hamiltonian comprised of a linear *H*_L_ and a nonlinear *H*_NL_ component, i.e., *H* = *H*_L_ + *H*_NL_ where ${H}_{{{{{{{{\rm{L}}}}}}}}}=\mathop{\sum }\nolimits_{m=1}^{M}({a}_{m}{a}_{m+1}^{*}+{a}_{m}^{*}{a}_{m+1})$ and ${H}_{{{{{{{{\rm{NL}}}}}}}}}=\mathop{\sum }\nolimits_{m=1}^{M}\frac{1}{2}|{a}_{m}{|}^{4}$, where *a*_*M*+1_ = 0 because of the truncated boundary condition. Under weak nonlinear conditions, the contribution from the linear term *H*_L_ dominates, and as a result one can define a quasi-invariant internal energy by $U\equiv -{H}_{{{{{{{{\rm{L}}}}}}}}}=-\mathop{\sum }\nolimits_{j=1}^{M}{\varepsilon }_{j}|{c}_{j}{|}^{2}$, where ${\varepsilon }_{j}=2\cos (\frac{j\pi }{M+1})$ are the eigenvalues associated with the linear supermodes $|{\psi }_{j}\rangle$. As indicated above, by using purely entropic principles, one can show that light propagating in such a system evolves towards a thermal state obeying the RJ distribution^34,35^:

2 $$|{c}_{j}{|}^{2}=-\frac{T}{\mu+{\varepsilon }_{j}},$$

where *T* and *μ* represents the optical temperature and chemical potential, respectively. In general, the equilibrium values of *T*, *μ* can be predicted from the initial conditions, i.e., from the invariants ${{{{{{{\mathcal{P}}}}}}}}$ and *U*^34,36,38^. For instance, for a lattice with *M* = 100 elements, an input excitation ∣*c*_*j*_∣^2^ = 0.05(*ε*_*j*_ + 2) (dashed line in Fig. 2b) leads to ${{{{{{{\mathcal{P}}}}}}}}=10$ and *U* = − 9.9, which in turn predicts *T* = 0.15 and *μ* = − 2.5 (see Supplementary Note 1). The size of the systems considered in this study is large enough so as to guarantee the extensivity of the entropy and the self-consistency of the thermodynamic formulation used^44^. By numerically integrating Eq. (1), we find that the equilibrium modal occupancies ∣*c*_*j*_∣^2^ are consistent with the theoretically predicted RJ distribution (Fig. 2b). The inset panel in Fig. 2b shows that during propagation, the optical entropy $S=\mathop{\sum }\nolimits_{j=1}^{M}\ln (|{c}_{j}{|}^{2})$ monotonically increases until it reaches a maximum (as expected by the second law of thermodynamics) while the optical energy *U* remains quasi-invariant.

### Cascade second order *χ*^(2)^ nonlinearity

In order to demonstrate the universality of RJ thermalization, we now investigate a variety of scenarios. In this respect, we consider cascade second order *χ*^(2)^ nonlinear processes unfolding in waveguide arrays, governed by the following normalized coupled evolution equations^45–47^:

3 $$\begin{array}{ll}&i\dfrac{d{a}_{m}}{dz}+{a}_{m-1}+{a}_{m+1}+{a}_{m}^{*}{b}_{m}=0,\\ &i\dfrac{d{b}_{m}}{dz}-{{\Delta }}{b}_{m}+{a}_{m}^{2}=0,\hfill\end{array}$$

where *a*_*m*_ and *b*_*m*_ are the local site field amplitudes associated with the fundamental and the second-harmonic frequency, and Δ is the phase mismatch. Here the linear coupling among *b*_*m*_ is neglected^46^, as illustrated in Fig. 2c. This system exhibits two constants of motion: the total optical power ${{{{{{{\mathcal{P}}}}}}}}=\mathop{\sum }\nolimits_{m=1}^{M}(|{a}_{m}{|}^{2}+|{b}_{m}{|}^{2})$ and the Hamiltonian $H=\mathop{\sum }\nolimits_{m=1}^{M}[{a}_{m}{a}_{m+1}^{*}+{a}_{m}^{*}{a}_{m+1}-\frac{1}{2}{{\Delta }}|{b}_{m}{|}^{2}+\frac{1}{2}({a}_{m}^{2}{b}_{m}^{*}+{a}_{m}^{*2}{b}_{m})]$ (see Supplementary Note 2). Under weak nonlinear conditions, the field in the fundamental frequency *a*_*m*_ dominates, and therefore its power and energy can be regarded as quasi-invariants, i.e., ${{{{{{{{\mathcal{P}}}}}}}}}_{a}=\mathop{\sum }\nolimits_{j=1}^{M}|{c}_{j}{|}^{2}$, and ${U}_{a}=-\mathop{\sum }\nolimits_{j=1}^{M}{\varepsilon }_{j}|{c}_{j}{|}^{2}$, where *c*_*j*_ is the field amplitude of the corresponding supermode . If indeed this system can thermalize through the *χ*^(2)^ process under these two invariants, one should then anticipate a RJ distribution once equilibrium is reached. To confirm this hypothesis, we numerically simulated Eq. (3) with Δ = 1, *M* = 100 when the first 30 modes in the fundamental frequency were evenly excited (dashed line in Fig. 2d). As shown in Fig. 2d, after a non-equilibrium prethermalization stage, the quantities ${{{{{{{{\mathcal{P}}}}}}}}}_{a}$ and *U*_*a*_ eventually settle to ${{{{{{{{\mathcal{P}}}}}}}}}_{a}=8.3$ and *U*_*a*_ = −15.1, i.e., they remain invariants. For this set of values, once thermal equilibrium is attained, our theory predicts *T* = 0.016 and *μ* = −2.007, in excellent agreement with our numerical simulations (Fig. 2d).

### Optomechanical nonlinearity

Next, we consider a lossless nonlinear optomechanical cavity array where the intracavity optical fields and the vibrational motions are described by the following evolution equations^48^:

4 $$\begin{array}{ll}&i\dfrac{d{a}_{m}}{dt}-({a}_{m-1}+{a}_{m+1})+{a}_{m}({b}_{m}+{b}_{m}^{*})=0,\\ &i\dfrac{d{b}_{m}}{dt}-{{\Omega }}{b}_{m}+|{a}_{m}{|}^{2}=0.\hfill\end{array}$$

Here *a*_*m*_ and *b*_*m*_ stands for the optical field and the mechanical oscillation amplitude in cavity *m*, respectively (Fig. 2e), while the parameter Ω represents a normalized angular frequency of the mechanical resonance. Synchronization between driven optomechanical oscillators have been investigated in earlier studies and it was shown that the synchronization dynamics follow the generic features of the Kuramoto model^49^. Here, instead, we are interested in the nonlinear dynamics of coupled optomechanical oscillators in the absence of the driving force. We proceed by first noting that the above system exhibits two invariants: the number of “photons” in the cavities ${{{{{{{{\mathcal{P}}}}}}}}}_{a}=\mathop{\sum }\nolimits_{m=1}^{M}|{a}_{m}{|}^{2}=\mathop{\sum }\nolimits_{j=1}^{M}|{c}_{j}{|}^{2}$, and the overall Hamiltonian of the system $H=\mathop{\sum }\nolimits_{m=1}^{M}[-({a}_{m}{a}_{m+1}^{*}+{a}_{m}^{*}{a}_{m+1})+|{a}_{m}{|}^{2}({b}_{m}+{b}_{m}^{*})-{{\Omega }}|{b}_{m}{|}^{2}]$ (see Supplementary Note 3), where *c*_*j*_ denotes the field amplitude of the *j*_th_ optical supermode. As before, under weakly nonlinear conditions and when the normalized Ω is large, such as Ω = 8 in our numerical simulations, one finds that the linear part of the Hamiltonian associated with the optical field is a quasi-invariant, ${U}_{a}=\mathop{\sum }\nolimits_{m=1}^{M}[-({a}_{m}{a}_{m+1}^{*}+{a}_{m}^{*}{a}_{m+1})]=\mathop{\sum }\nolimits_{j=1}^{M}{\varepsilon }_{j}|{c}_{j}{|}^{2}$. Even in this more complex scenario, the RJ distribution emerges at thermal equilibrium as a result of ergodicity as can be seen in Fig. 2f. In all cases, a good agreement was found to exist between numerical simulations and the theoretically anticipated RJ distribution once ${{{{{{{{\mathcal{P}}}}}}}}}_{a}$, *U*_*a*_ were specified by initial conditions. Note that in this case, it is impossible to associate a multi-wave mixing process to the optical nonlinearity—an aspect that dispels the wave turbulence paradigm. Interestingly, unlike their photon counterparts, the mechanical vibrations themselves do not display a pair of (quasi-)invariants ${{{{{{{\mathcal{P}}}}}}}}$ and *U* (see Supplementary Note 3), and therefore cannot thermalize to a RJ equilibrium state in the same manner.

### Nonlinearity described by a smooth but nowhere analytic function

So far, we have analyzed thermalization effects in multimode systems where the nonlinearities conform to standard Taylor series expansions. Naturally, one may ask whether the RJ thermalization process can indeed manifest itself in more general nonlinear settings. To address this question, we now consider optical lattices involving generalized intensity-dependent nonlinearities *F*(*x*) as described by^50^:

5 $$i\frac{d{a}_{m}}{dz}+{a}_{m-1}+{a}_{m+1}+F(|{a}_{m}{|}^{2}){a}_{m}=0.$$

Here the optical power ${{{{{{{\mathcal{P}}}}}}}}=\mathop{\sum }\nolimits_{j=1}^{M}|{c}_{j}{|}^{2}$ as well as the Hamiltonian $H=\mathop{\sum }\nolimits_{m=1}^{M}[{a}_{m}{a}_{m+1}^{*}+{a}_{m}^{*}{a}_{m+1}+G(|{a}_{m}{|}^{2})]$ of the system are still conserved, where *G*(*x*) is the antiderivative of *F*(*x*) (i.e., *dG*(*x*)/*dx* = *F*(*x*), and *G*(0) = 0). As before, in the weak nonlinear regime, i.e., *F*(*x*) ≪ 1, the linear part of the Hamiltonian $U=-\mathop{\sum }\nolimits_{j=1}^{M}{\varepsilon }_{j}|{c}_{j}{|}^{2}$ is a quasi-invariant.

**Fig. 3:**
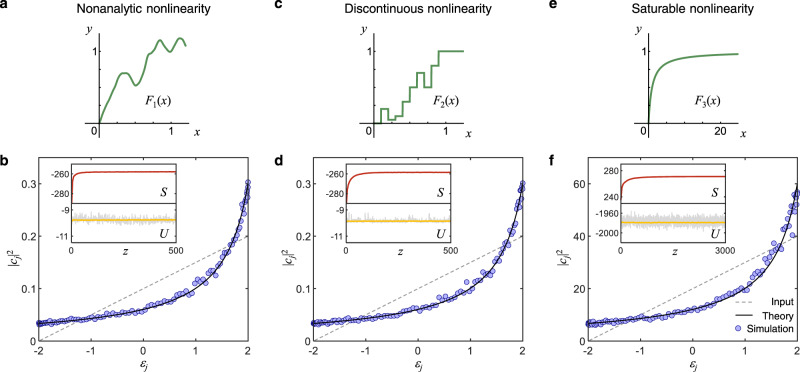
Thermalization of light in nonlinear lattices involving generalized intensity-dependent nonlinearities *F*(*x*). **a** An example of non-analytic function used in our simulations. **b** Corresponding Rayleigh–Jeans (RJ) distribution (*T* = 0.15, *μ* = −2.5) occurring after thermalization. **c** A discontinuous multi-step function used in our simulations. **d** Again this nonlinearity leads to a RJ distribution. **e** A saturable nonlinearity described by ${F}_{3}(x)=\frac{x}{1+x}$, and (**f**) its corresponding RJ distribution. In (b) and (d), the initial excitation conditions are exactly the same and as a result they attain the same RJ allocation, an aspect indicating universality in thermalization.The insets have been plotted in a manner similar to Fig. 2. As before, here we used *M* = 100 and the initial mode occupancies are represented by the dashed lines.

First, we consider the case where *F*(*x*) is chosen to be a smooth (infinitely differentiable) function everywhere, yet nowhere analytic (i.e., it does not have a convergent Taylor series representation). This function, which we will henceforth denote as *F*_1_(*x*). For example, here we construct such a nonanalytic function via Fourier series ${F}_{1}(x)=\mathop{\sum }\nolimits_{n=-N}^{N}{h}_{n}\exp (i2\pi nx)$, where the Fourier coefficients *h*_*n*_ are random variables chosen such that their amplitudes drop with *n* faster than the reciprocal of any polynomial but slower than exponential^51–53^ (see Supplementary Note 4). This condition guarantees that in the limit *N* → *∞*, the function *F*_1_(*x*) is infinitely differentiable but nowhere analytic. In other words, this function has a Taylor series but its radius of convergence tends to 0 as *N* → *∞*. From a practical point of view, one can choose *N* to be large enough so as the function *F*_1_(*x*) does not have a proper Taylor series within the range of interest of the intensities involved in our simulations. Figure 3a shows one such possible function *F*_1_(*x*) used in our computations. In this case, numerical simulations carried out on Eq. (5) clearly indicate that the RJ distribution still emerges upon thermalization, as shown in Fig. 3b. While these results clearly support the universality hypothesis for RJ thermalization, they still do not provide compelling evidence, mainly because the function *F*_1_(*x*) is continuous. In this case, the Stone–Weierstrass theorem^54^ guarantees that it can be still represented by a polynomial expansion, even though it does not correspond to its Taylor series. Thus, in this scenario one could still argue that the underlying nonlinear interactions do arise from a series of higher-order wave mixing terms.

### A discontinuous nonlinearity function

In order to assert the universality of RJ thermalization, i.e., being of a purely entropic (ergodic) origin that goes beyond the wave mixing picture, we next consider a nonlinearity that is described by a discontinuous multi-step function^55–57^ such as that depicted in Fig. 3c, denoted as *F*_2_(*x*). Due its discontinuous nature, the function *F*_2_(*x*) cannot be analytically represented by a polynomial expansion across its entire domain. In other words, the wave mixing paradigm completely fails in this case. Interestingly, even in this case, the system thermalizes and reaches a RJ equilibrium state as shown in Fig. 3d, in full accord with theoretically anticipated results. This latter example demonstrates once and for all that optical thermalization in multimode systems has a more fundamental origin—rooted in the system’s ergodicity rather than in the intricate nature of the nonlinear interactions involved. In other words, the onset of a RJ distribution does not necessarily require the presence of any multi-wave mixing mechanisms. Instead, it is simply the outcome of the maximizing the entropy itself. Note that the simulations depicted in Fig. 3b, d were carried out for the same parameters and initial conditions (*M* = 100, ${{{{{{{\mathcal{P}}}}}}}}=10$, *U* = −9.9). Interestingly, despite the profound differences in their nonlinearity, they all settle exactly at the same RJ distribution with *T* = 0.15 and *μ* = −2.5. This further supports our hypothesis. In other words, as indicated before, one cannot infer the nature of the intermolecular collision processes from the Maxwell–Boltzmann distribution as manifested in actual gases.

### Saturable nonlinearity

We finally extend this discussion to more realistic material systems. For instance, consider photorefractive crystals where the nonlinearity is saturable^57,58^ ${F}_{3}(x)=\frac{x}{1+x}$, as shown in Fig. 3e. In the domain where *x* > 1, *F*_3_(*x*) does not have a Taylor representation but instead has a Laurent series expansion^59^: *F*_3_(*x*) = 1 − *x*^−1^ + *x*^−2^ − *x*^−3^ + . . . . Obviously, in this regime, the nonlinear interaction cannot be described by a simple wave mixing approach. Yet, assuming that ergodicity holds, and given that two invariants ${{{{{{{\mathcal{P}}}}}}}}$ and *U* still exist, as per our previous arguments, this should lead to RJ thermalization. This is verified using numerical simulations as shown in Fig. 3f. To ensure the validity of our conclusions, the values of the local intensities ∣*a*_*m*_∣^2^ have been monitored during our simulations so as the *F*_3_(*x*) function was predominantly within the Laurent series expansion (see Supplementary Note 5).

## Discussion

In conclusion, we have critically examined the manner in which optical thermalization processes unfold in nonlinear multimode environments and showed that the RJ distribution law is universal: it can manifest itself even in systems where the multi-wave mixing picture fails. These results extend the notion of wave thermalization beyond the original wave turbulence hypothesis that is founded on the premise of wave mixing interactions. In other words, through the use of counterexamples we demonstrated that nonlinear wave mixing may be sufficient but by no means necessary. Importantly, it would seem that, in some cases, these processes may not be in fact responsible for thermalization. Instead, our results suggest that RJ equilibrium is obtained because of ergodicity and entropy maximization as expected by the second law of thermodynamics. These observations, not only support a thermodynamic/probabilistic interpretation of these results, but also provide appropriate foundations to expand the thermodynamic formalism in other physical settings governed by classical bosonic interactions. Finally, of interest would be to investigate the prospects of devising a formal proof that would dictate the universality of thermalization processes under general nonlinear conditions.

## Methods

### Numerical simulation

All the simulation results in this work are obtained by numerically integrating the nonlinear equations of motion described by Eqs. (1), and (3)–(5). Due to the finite size of the system, the modal occupancies ∣*c*_*j*_∣^2^ fluctuate around their equilibrium values. Thus, the final equilibrium state can be evaluated either by calculating the time (distance) average or ensembles average. In this work, we adopted the latter strategy. In particular, for each simulation in Figs. 2 and 3, we have employed 400 ensembles, each of which corresponds to a random initial condition that have the same intensity profile (i.e., same values for ∣*c*_*j*_∣^2^) but a different phase distribution.

## Supplementary information

Supplementary Information

## Data Availability

The data that support the findings of this study are available from the corresponding authors upon request.
