# Supplementary material for: Universality of light thermalization in multimoded nonlinear optical systems
Source: Nat Commun. 2023 Jan 23;14:370. doi: 10.1038/s41467-023-35891-9 (PMC9871037; doi:10.1038/s41467-023-35891-9)
Supplement: Supplementary file 1 — Supplementary Information [file 41467_2023_35891_MOESM1_ESM.pdf]

Universality of light thermalization in multimoded nonlinear optical systems
Supplementary Information

Qi Zhong,[1,*] Fan O. Wu,[1,*] Absar U. Hassan,[1] Ramy El-Ganainy,[2,3,†] and Demetrios N. Christodoulides[1,4,‡]

[1] *CREOL, College of Optics and Photonics, University of Central Florida, Orlando, FL 32816, USA*

[2] *Department of Physics, Michigan Technological University, Houghton, MI 49931, USA*

[3] *Henes Center for Quantum Phenomena, Michigan Technological University, Houghton, MI 49931, USA*

[4] *Ming Hsieh Department of Electrical and Computer Engineering, University of Southern California, Los Angeles, CA 90089, USA*

* These two authors contributed equally to this work.

† ganainy@mtu.edu

‡ demetri@creol.ucf.edu

Supplementary Note 1: Calculation of temperature T and chemical potential μ

Generally, for an arbitrary nonlinear photonic lattice, the temperature T and chemical potential μ after reaching the equilibrium can be numerically predicted by the initial condition $\mathcal{P}$, U, M and the spectrum ε_j [1, 2]. A recent work [3] shows that, under large M condition, explicit formulas of T and μ can be obtained for a one-dimensional photonic array with uniform nearest-neighbour coupling κ, and that is

$$\frac{1}{T} = \frac{2MU}{U^2 - 4\kappa^2\mathcal{P}^2}, \tag{1.1}$$

$$\frac{\mu}{T} = \frac{M}{\mathcal{P}} + \frac{8M\kappa^2\mathcal{P}}{U^2 - 4\kappa^2\mathcal{P}^2}. \tag{1.2}$$

Rewriting Eqs. (1.1) and (1.2) can get

$$T = \frac{U^2 - 4\kappa^2\mathcal{P}^2}{2MU}, \tag{1.3}$$

$$\mu = \frac{U^2 + 4\kappa^2\mathcal{P}^2}{2\mathcal{P}U}. \tag{1.4}$$

Table 1 list the values of $\mathcal{P}$ and U used in the simulations in Fig. 2 and Fig. 3 of the main text and the predicted values of T and μ according to Eqs. (1.3) and (1.4). In all simulations, we take $\kappa = 1$ and $M = 100$.

Simulation	$\mathcal{P}$	U	T	μ
Fig. 2b	10	−9.9	0.15	−2.5
Fig. 2d	8.3	−15.1	0.016	−2.009
Fig. 2f	50	−49.5	0.76	−2.5
Fig. 3b	10	−9.9	0.15	−2.5
Fig. 3d	10	−9.9	0.15	−2.5
Fig. 3f	2000	−1980	30.5	−2.5

TABLE I. The values of $\mathcal{P}$ and U used in simulations in Figs. 2 and 3 of the main text and the calculated value of T and μ based on Eqs. (1.3) and (1.4).

Supplementary Note 2: Conservation laws in cascade $\chi^{(2)}$ process

The governing equation for cascade $\chi^{(2)}$ process is

$$\begin{aligned} i\frac{da_m}{dz} + a_{m-1} + a_{m+1} + a_m^* b_m = 0, \\ i\frac{db_m}{dz} - \Delta b_m + a_m^2 = 0, \end{aligned} \tag{2.1}$$

where a_m and b_m is the field amplitudes associated with the fundamental frequency and its second-harmonic on the mth site, respectively, and Δ is the phase mismatch. The associated Hamiltonian can be

$$H = \sum_{m=1}^{M} \left[a_m a_{m+1}^* + a_m^* a_{m+1} - \frac{1}{2}\Delta |b_m|^2 + \frac{1}{2}(a_m^2 b_m^* + a_m^{*2} b_m) \right]. \tag{2.2}$$

By introducing the generalized coordinates and momenta coordinates $(a_m, \pi_m, b_m, \tau_m)$, where $\pi_m = -ia_m^*$ and $\tau_m = -\frac{i}{2}b_m^*$, the Hamiltonian in Eq. (2.2) can be written as

$$H = \sum_{m=1}^{M} \left(ia_m \pi_{m+1} + ia_{m+1}\pi_m - i\Delta b_m \tau_m + ia_m^2 \tau_m - \frac{1}{2}\pi_m^2 b_m \right). \tag{2.3}$$

It is easy to check that this Hamiltonian can recover the dynamic equation described by Eq. (2.1) by following

$$\begin{cases} \dfrac{da_m}{dz} = \dfrac{\partial H}{\partial \pi_m}, \dfrac{d\pi_m}{dz} = -\dfrac{\partial H}{\partial a_m} \\ \dfrac{db_m}{dz} = \dfrac{\partial H}{\partial \tau_m}, \dfrac{d\tau_m}{dz} = -\dfrac{\partial H}{\partial b_m}. \end{cases} \tag{2.4}$$

The Hamiltonian H does not explicitly depend on z ($\frac{\partial H}{\partial z} = 0$), and

$$\frac{dH}{dz} = [H, H] + \frac{\partial H}{\partial z} = 0 + 0 = 0, \tag{2.5}$$

therefore H is conserved. Here the Poisson bracket is defined as:

$$[A, B] = \sum_i \left(\frac{\partial A}{\partial q_i}\frac{\partial B}{\partial p_i} - \frac{\partial A}{\partial p_i}\frac{\partial B}{\partial q_i} \right), \tag{2.6}$$

where q_i and p_i are the generalized coordinates and canonical momenta.

The second conservation law in this system is $\mathcal{P} = \sum_{m=1}^{M}(|a_m|^2 + |b_m|^2) = \sum_{m=1}^{M}(ia_m\pi_m +$

$2ib_m\tau_m$). This can be checked by the Poisson bracket

$$
\begin{aligned}
[\mathcal{P}, H] &= \sum_{m=1}^{M} \left(\frac{\partial \mathcal{P}}{\partial a_m}\frac{\partial H}{\partial \pi_m} - \frac{\partial \mathcal{P}}{\partial \pi_m}\frac{\partial H}{\partial a_m} + \frac{\partial \mathcal{P}}{\partial b_m}\frac{\partial H}{\partial \tau_m} - \frac{\partial \mathcal{P}}{\partial \tau_m}\frac{\partial H}{\partial b_m} \right) \\
&= \sum_{m=1}^{M} \big[i\pi_m(ia_{m-1} + ia_{m+1} - \pi_m b_m) - ia_m(i\pi_{m+1} + i\pi_{m-1} + 2ia_m\tau_m) \\
&\qquad + 2i\tau_m(-i\Delta b_m + ia_m^2) - 2ib_m(-i\Delta\tau_m - \frac{1}{2}\pi_m^2) \big] \\
&= 0.
\end{aligned} \tag{2.7}
$$

Therefore

$$\frac{d\mathcal{P}}{dz} = [\mathcal{P}, H] + \frac{\partial \mathcal{P}}{\partial z} = 0 + 0 = 0. \tag{2.8}$$

Supplementary Note 3: Conservation laws in nonlinear optomechanical cavities

The governing equation for a nonlinear optomechanical cavity array is

$$
\begin{aligned}
&i\frac{da_m}{dt} - (a_{m-1} + a_{m+1}) + a_m(b_m + b_m^*) = 0, \\
&i\frac{db_m}{dt} - \Omega b_m + |a_m|^2 = 0,
\end{aligned} \tag{3.1}
$$

where a_m and b_m stands for the optical field and the mechanical oscillation amplitude in cavity m, respectively. Here the parameter Ω represents the frequency detuning. The Hamiltonian is

$$H = \sum_{m=1}^{M} \left[-(a_m a_{m+1}^* + a_m^* a_{m+1}) + |a_m|^2(b_m + b_m^*) - \Omega|b_m|^2 \right]. \tag{3.2}$$

The coordinates and canonical momenta are $(a_m, \pi_m, b_m, \tau_m)$ and $\pi_m = -ia_m^*$, $\tau_m = -ib_m^*$. Then the Hamiltonian can be written as

$$H = \sum_{m=1}^{M} \left[-i(a_m\pi_{m+1} + a_{m+1}\pi_m) + ia_m\pi_m(b_m + i\tau_m) - i\Omega b_m\tau_m \right]. \tag{3.3}$$

The Hamiltonian H in Eq. (3.3) can recover the dynamic equation described in Eq. (3.1) and it is conserved since it does not depend on t explicitly. The second conservation law is

$\mathcal{P}_a = \sum_{m=1}^{M} |a_m|^2 = \sum_{m=1}^{M} ia_m\pi_m$, since the Poisson bracket $[\mathcal{P}_a, H] = 0$ as follows:

$$\begin{aligned}
[\mathcal{P}_a, H] &= \sum_{m=1}^{M} \left(\frac{\partial \mathcal{P}_a}{\partial a_m} \frac{\partial H}{\partial \pi_m} - \frac{\partial \mathcal{P}_a}{\partial \pi_m} \frac{\partial H}{\partial a_m} + \frac{\partial \mathcal{P}_a}{\partial b_m} \frac{\partial H}{\partial \tau_m} - \frac{\partial \mathcal{P}_a}{\partial \tau_m} \frac{\partial H}{\partial b_m} \right) \\
&= \sum_{m=1}^{M} \{ i\pi_m[-ia_{m-1} - ia_{m+1} + ia_m(b_m + i\tau_m)] - ia_m[-i\pi_{m+1} - i\pi_{m-1} + i\pi_m(b_m + i\tau_m)] \} \\
&= 0.
\end{aligned} \tag{3.4}$$

On the other hand, $\mathcal{P}_b = \sum_{m=1}^{M} |b_m|^2 = \sum_{m=1}^{M} ib_m\tau_m$ for the mechanical oscillators is not conserved, since $[\mathcal{P}_b, H] \neq 0$ as follows:

$$\begin{aligned}
[\mathcal{P}_b, H] &= \sum_{m=1}^{M} \left(\frac{\partial \mathcal{P}_b}{\partial a_m} \frac{\partial H}{\partial \pi_m} - \frac{\partial \mathcal{P}_b}{\partial \pi_m} \frac{\partial H}{\partial a_m} + \frac{\partial \mathcal{P}_b}{\partial b_m} \frac{\partial H}{\partial \tau_m} - \frac{\partial \mathcal{P}_b}{\partial \tau_m} \frac{\partial H}{\partial b_m} \right) \\
&= \sum_{m=1}^{M} [i\tau_m(-a_m\pi_m - i\Omega b_m) - ib_m(ia_m\pi_m - i\Omega\tau_m)] \\
&= \sum_{m=1}^{M} i|a_m|^2(b_m^* - b_m) \\
&\neq 0.
\end{aligned} \tag{3.5}$$

Supplementary Note 4: Smooth but nonanalytic function

A smooth non-analytic function $f_1(x)$ can be generated numerically by using the method described in Ref. [4]. One example of such type of functions is shown in Fig. S1a. This function is nowhere analytic in its domain since its Taylor series expansion does not converge. This is illustrated in Fig. S1b, where we observe that the magnitude of the first, second and third derivatives of $f_1(x)$ grows from 50 to the order of 10^6. In our study of light thermalization, we considered only a part of this f_1 (see red line in Fig. S1a) and performed the following transformation to obtain $F_1(x)$ shown in Fig. S1c):

$$F_1(x) = \frac{f_1[(x_2 - x_1)x + x_1] - y_1}{y_2 - y_1}, \tag{4.1}$$

where $x_1 = 0.25$, $x_2 = 0.5$, and $y_1 = f_1(x_1) \approx -1.34$, $y_2 = f_1(x_2) \approx 1.27$. This re-normalization ensures that $F_1(0) = 0$, $F_1(1) = 1$ and $F_1(x) > 0$ in the domain of $[0, 1]$.

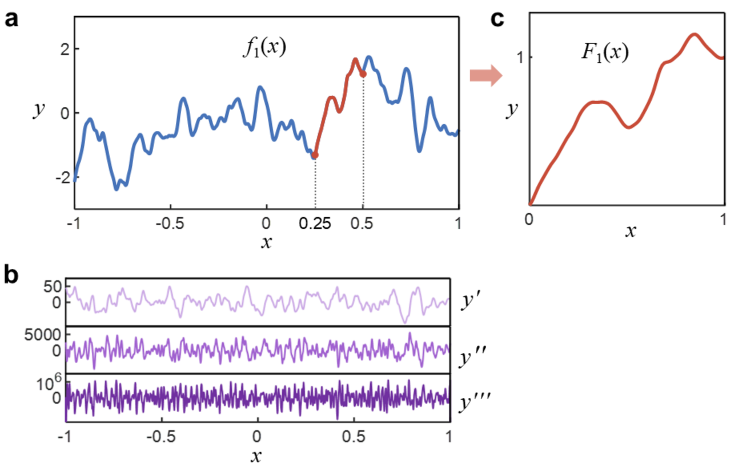

FIG. S1. **a** The smooth everywhere but nowhere analytic function $f_1(x)$ constructed by Fourier series with random coefficients of root-exponentially decreasing amplitudes. **b** The first, second and third derivatives of function $f_1(x)$. **c** In our simulation, we use a function $F_1(x)$, which is a part of $f_1(x)$ after a transformation according to Eq. (4.1).

Supplementary Note 5: Field intensity distribution in the saturable nonlinearity

In the simulation of a saturable nonlinearity in Fig. 3f of the main text, to ensure that the Laurent series expansion is valid (i.e., $|a_m|^2 > 1$), the values of the local intensities $|a_m|^2$ have been monitored, and their distribution is illustrated in Fig. S2. As we can see, only 5% of $|a_m|^2$ locate in the range of $[0, 1]$ and the rest are greater than unity .

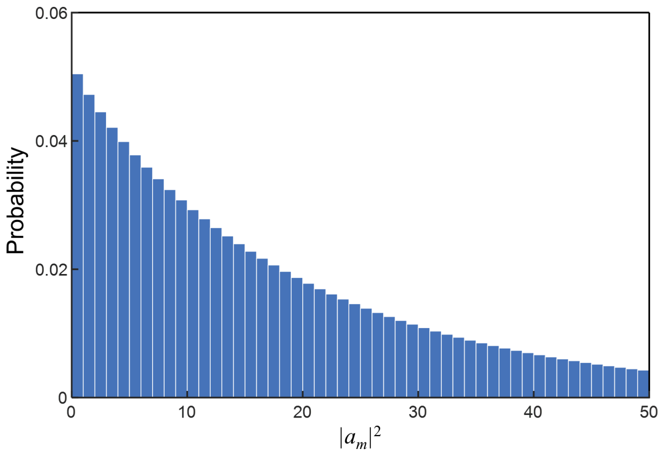

FIG. S2. The distribution of the values of $|a_m|^2$ during simulation.

Supplementary Note 6: Thermalization in a SSH lattice

The light thermalization is a general phenomenon, which can happen in a lattice with uniform coupling as discussed in the main text, but also can occur in topological lattice with nonuniform coupling coefficients. In this section, we investigate the optical thermalization in a topological Su–Schrieffer–Heeger(SSH) lattice (Fig. S3a). The coupling coefficients are taken $\kappa_1 = 4/3$, $\kappa_2 = 2/3$, and the coresponding spectrum ε_j is plotted in Fig. S3b. In this lattice, an input excitation $|c_j|^2 = 0.05(\varepsilon_j + 2)$ (dashed line in Fig. S3c) leads to $\mathcal{P} = -10.9$, which in turn predicts the RJ distributation with $T = 0.13$ and $\mu = -2.4$, which is consitent with the numerical simulation result as shown in Fig. S3c.

Moreover, a non-Hermitian SSH model with asymmetric couplings between nearest neighbors was reported in [5] recently.

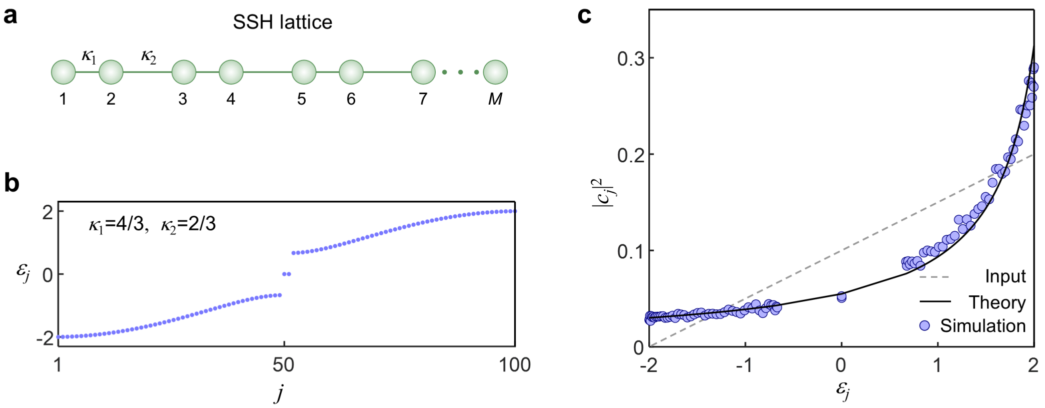

FIG. S3. **a** A Su–Schrieffer–Heeger (SSH) lattice lattice with M sites. The coupling coefficients are κ_1 and κ_2, respectively. **b** The spectrum ε_j under the condition that $\kappa_1 = 4/3$, $\kappa_2 = 2/3$ and $M = 100$. **c** An input excitation $|c_j|^2 = 0.05(\varepsilon_j + 2)$ (dashed line) leads to a RJ distribution with $T = 0.13$ and $\mu = -2.4$ (solid line), which is consistent with the numerical simulation result (blue dots).

Supplementary References

[1] F. O. Wu, A. U. Hassan, and D. N. Christodoulides, Nat. Photonics **13**, 776 (2019).

[2] M. Parto, F. O. Wu, P. S. Jung, K. Makris, and D. N. Christodoulides, Opt. Lett. **44**, 3936 (2019).

[3] F. O. Wu, P. S. Jung, M. Parto, M. Khajavikhan, and D. N. Christodoulides, Commun. Phys. **3**, 216 (2020).

[4] N. Trefethen, "Smoothies: nowhere analytic functions," https://www.chebfun.org/examples/stats/Smoothies.html (2020).

[5] G. G. Pyrialakos, H. Ren, P. S. Jung, M. Khajavikhan, and D. N. Christodoulides, Phys. Rev. Lett. **128**, 213901 (2022).
